# Color-coded summation images for the evaluation of blood flow in endovascular aortic dissection fenestration

**DOI:** 10.1186/s12880-022-00744-2

**Published:** 2022-02-04

**Authors:** Anne Marie Augustin, Franziska Wolfschmidt, Thilo Elsässer, Alexander Sauer, Alexander Dierks, Thorsten Alexander Bley, Ralph Kickuth

**Affiliations:** 1 Department of Diagnostic and Interventional Radiology, University Hospital Würzburg, Oberdürrbacher Strasse 6, 97080 Würzburg, Germany; 2 Department of Neuroradiology, Ludwigsburg Hospital, Ludwigsburg, Germany; 3 Siemens Healthcare GmbH, Forchheim, Germany; 4 BAG Radiologie Und Nuklearmedizin Aschaffenburg, Aschaffenburg, Germany; 5 Nuclear Medicine, Medical Faculty, University of Augsburg, Augsburg, Germany

**Keywords:** Aortic dissection, Angiography, Color-coding, DSA, Endovascular, Fenestration

## Abstract

**Background:**

To analyze the benefit of color-coded summation images in the assessment of target lumen perfusion in patients with aortic dissection and malperfusion syndrome before and after fluoroscopy-guided aortic fenestration.

**Methods:**

Between December 2011 and April 2020 25 patients with Stanford type A (n = 13) or type B dissection (n = 12) and malperfusion syndromes were treated with fluoroscopy-guided fenestration of the dissection flap using a re-entry catheter. The procedure was technically successful in 100% of the cases and included additional iliofemoral stent implantation in four patients. Intraprocedural systolic blood pressure measurements for gradient evaluation were performed in 19 cases. Post-processed color-coded DSA images were obtained from all DSA series before and following fenestration. Differences in time to peak (dTTP) values in the compromised aortic lumen and transluminal systolic blood pressure gradients were analyzed retrospectively. Correlation analysis between dTTP and changes in blood pressure gradients was performed.

**Results:**

Mean TTP prior to dissection flap fenestration was 6.85 ± 1.35 s. After fenestration, mean TTP decreased significantly to 4.96 ± 0.94 s (p < 0.001). Available systolic blood pressure gradients between the true and the false lumen were reduced by a median of 4.0 mmHg following fenestration (p = 0.031), with significant reductions in Stanford type B dissections (p = 0.013) and minor reductions in type A dissections (p = 0.530). A moderate correlation with no statistical significance was found between dTTP and the difference in systolic blood pressure (r = 0.226; p = 0.351).

**Conclusions:**

Hemodynamic parameters obtained from color-coded DSA confirmed a significant reduction of TTP values in the aortic target lumen in terms of an improved perfusion in the compromised aortic region. Color-coded DSA might thus be a suitable complementary tool in the assessment of complex vascular patterns prevailing in aortic dissections, especially when blood pressure measurements are not conclusive or feasible.

## Introduction

Acute aortic dissection is a severe and life-threatening subtype of acute aortic syndrome that is characterized by significant mortality rates at 48 h [1]. Beside cardiac sequelae, malperfusion syndromes are the most common complications secondary to aortic dissections, leading to significantly increased mortality rates [2–4].

Endovascular aortic fenestration is an established minimally invasive approach that might be the treatment of choice in patients with severe malperfusion syndromes with or without previous aortic repair surgery [2]. By puncture of the intimal flap, a re-entry is created, resulting in an outflow from the false lumen and a reduction in the transluminal blood pressure gradient [5].

Intravascular pressure measurements in both lumina might provide important information about the severity of pressure gradients before fenestration and the extent of hemodynamic equalization after the intervention [6]. Nevertheless, the evaluation by intraluminal manometry might be unfeasible or unreliable in some cases. This might particularly be the case if the extent of the true lumen collapse impedes sufficient blood pressure measurement, leading to inconclusive or unaltered pressure gradients [7].

Color-coded DSA is a post-processing technique that enables the visualization of hemodynamic information of a monochromatic DSA series within a single composite image. While its benefit has been demonstrated in the field of neuroradiological and peripheral artery interventions, its application in the context of aortic flap fenestration in aortic dissections has so far not been reported [8, 9]. This study retrospectively evaluates the utilization of color-coded DSA imaging in patients with malperfusion syndromes due to aortic dissection, that has been treated with percutaneous endoluminal aortic fenestration.

## Materials and methods

### Study cohort

A review of the archives in our interventional radiology division resulted in 25 patients (13 men, 12 women) with aortic dissection and malperfusion syndromes who were consecutively treated by endovascular aortic fenestration between December 2011 and April 2020. 10 patients of the study population had been reported in another study series, in which the technique of fluoroscopy-guided balloon fenestration using a re-entry catheter was evaluated [10].

All Stanford type A dissections had been treated with thoracic aortic repair surgery before an endovascular fenestration procedure was conducted. Dissections were classified as acute in 24 cases and chronic in one case with presence of aortic dissection for 11 months prior to the procedure. The chronic type B dissection was iatrogenic and occurred during percutaneous coronary intervention. All other dissections occurred spontaneously. All included patients were treated as part of routine care and gave informed consent for the procedure. The local institutional review board waived its approval before conducting this retrospective study. All patients received cross-sectional imaging before the intervention, including computed tomography angiography (CTA) in 22 cases and magnetic resonance angiography (MRA) in three cases.

Branch vessel affection resulted in mesenterial (n = 4), renal (n = 5), spinal (n = 4) and iliofemoral malperfusion syndrome (n = 6). In six cases, a combination of different malperfusion syndromes was present, including mesenterial and spinal malperfusion in two cases, renal and iliofemoral malperfusion in two cases and spinal, renal and iliofemoral malperfusion in two cases. Clinical symptoms of spinal malperfusion were ischemic monoplegia or paraplegia. Signs of renal malperfusion were renal failure with high serum creatinine levels. Mesenterial malperfusion was associated with abdominal pain and elevated serum lactate levels and iliofemoral malperfusion with pain, pulselessness, paraesthesia, and poikilothermia of the lower extremities. Patient characteristics and procedural data are given in Table 1.

**Table 1:** Patients and procedural data

Mean age (years)	58.8 ± 11.3	
Male:female ratio	13:12	

	n	%
*Standford type*		
A	13	52.0
B	12	48.0
Acute	1	4.0
Chronic	24	96.0
Iatrogene	1	4.0
*Malperfusion syndrome*		
Spinal	4	16.0
Mesenterial	4	16.0
Renal	5	20.0
Iliofemoral	6	24.0
Combined	6	24.0
*Fenestration windows*		
1	16	64.0
2	7	28.0
3	2	8.0
*Fenestration direction*		
False towards true lumen	22	88.0
True toward false lumen	3	12.0
*Fenestration level*		
Infradiaphragmal	2	8.0
Mesenterial	8	32.0
Renal	8	32.0
Infrarenal	2	8.0
Aortic bifurcation	11	44.0
*Previous surgery*		
Replacement of ascending Aorta	13	52.0
Stent graft implantation	1	4.0
Subsequent iliofemoral stenting	4	16.0
Technical success	25	100

### Fenestration procedure

All procedures were performed by the same experienced board-certified operator in our local angiography suite (Axiom Artis Zee, Siemens Healthcare GmbH, Forchheim, Germany). Vascular access depended on the extension of the dissection membrane into the iliofemoral vasculature based on cross-sectional studies. In 14 cases, vascular access was created via the right common femoral artery and in six cases via the left common femoral artery. In five cases, a bilateral femoral access was utilized. All procedures were performed under local anaesthesia (Scandicain 1%, AstraZeneca; Cambridge, UK). A 6 – 7F-sheath (Terumo, Tokyo, Japan) was positioned within the true lumen whenever possible. For the acquisition of aortography, a pigtail-shaped catheter was placed in the aorta proximal to the level of maximum true lumen compromise. DSA parameters were: tube potential 70 kVp, body weight adapted tube current setting, 7.5 pulses per second, and a 480 mm field of view. In general, a 1:1 iodinated contrast medium (300 mg/ml)/saline dilution was applied.

In all procedures, percutaneous balloon fenestration of the dissection membrane was performed utilizing a fluoroscopy-based re-entry catheter (Outback LTD; Cordis, Miami Lakes, Fla), as reported elsewhere [10].

Systolic blood pressure measurements in the true and false lumen were introduced routinely during the study period and thus were not available in the first five patients of the study. In one patient, manometry was technically not feasible due to failure of the device. In total, complete systolic blood pressure values were available in 19 cases. Sufficient equalization between both lumina was defined as a pressure gradient after fenestration of 5 mmHg or less.

The puncture direction depended on the degree of true lumen collapse. The favoured target lumen was the false lumen, with puncture direction from the true lumen towards the false lumen in 22 cases. In the remaining three cases, puncturing from the false lumen towards the true lumen was necessary because of the minor extent of true lumen compromise. When flow equalization and reduction of true lumen collapse were insufficient after the first dissection flap fenestration, the fenestration process was repeated. In this regard, two fenestration windows were installed in seven cases, and three fenestration windows in two cases. Subsequent iliofemoral stent implantation was performed in four cases in which compromise of iliofemoral blood supply persisted after successful fenestration.

### Generation of color-coded images and data analysis

The acquired DSA series were transferred to the local picture archiving and communication system (PACS) (Syngo Plaza®, Siemens Healthcare GmbH, Erlangen, Germany and Merlin, Phoenix PACS®, Freiburg, Germany). The generation of post-processed color-coded images was performed utilizing the commercially available software Syngo iFlow® (Siemens Healthcare GmbH, Erlangen, Germany). This software enables the demonstration of hemodynamic information extracted from a monochromatic DSA series as a single color-coded composite image [11]. Blood flow characteristics are then expressed with different colors based on the time to peak (TTP), representing the time from contrast administration to the maximal contrast density in the vessel, with dark blue indicating delayed blood flow and red representing faster flow. A region of interest (ROI) was placed in the color-coded image and a time-contrast intensity curve as well as perfusion parameters such as time to peak (TTP) and area under the curve (AUC) were obtained automatically.

**Fig. 1:**
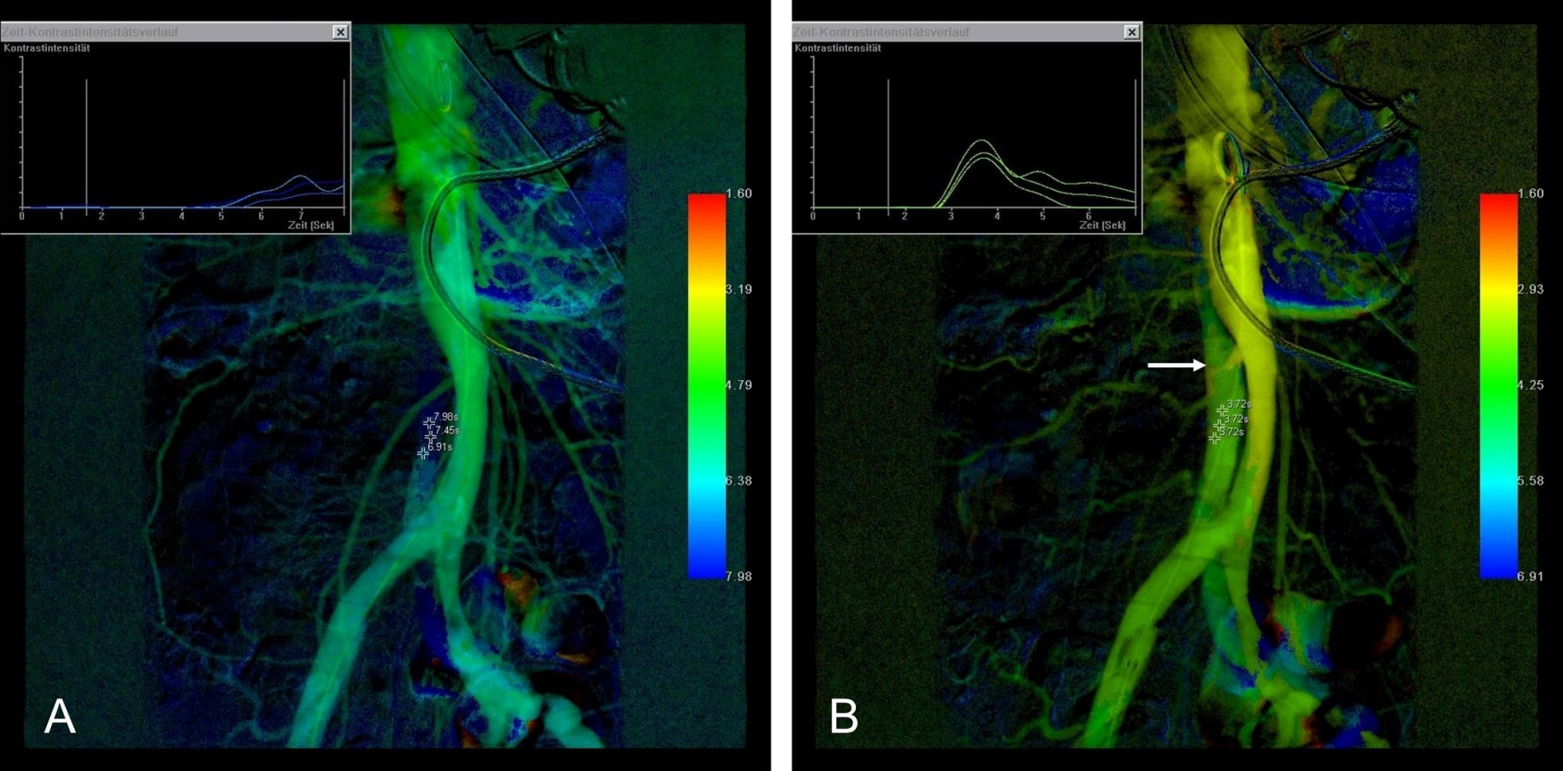
54-year-old male patient with spinal malperfusion syndrome due to acute Stanford type B aortic dissection. **a** Color-coded image gained from DSA prior to fenestration with ROI placement in the true lumen at the level of the infrarenal abdominal aorta. Compromised blood flow in the true lumen is represented by cold color gradients and corresponding flow curves (right corner). **b** After creation of two fenestration windows, the color-coded summation image demonstrates warmer color gradients at the measuring points and shortened TTP values. The arrow marks one of the fenestration windows just below the level of the renal artery

**Fig. 2:**
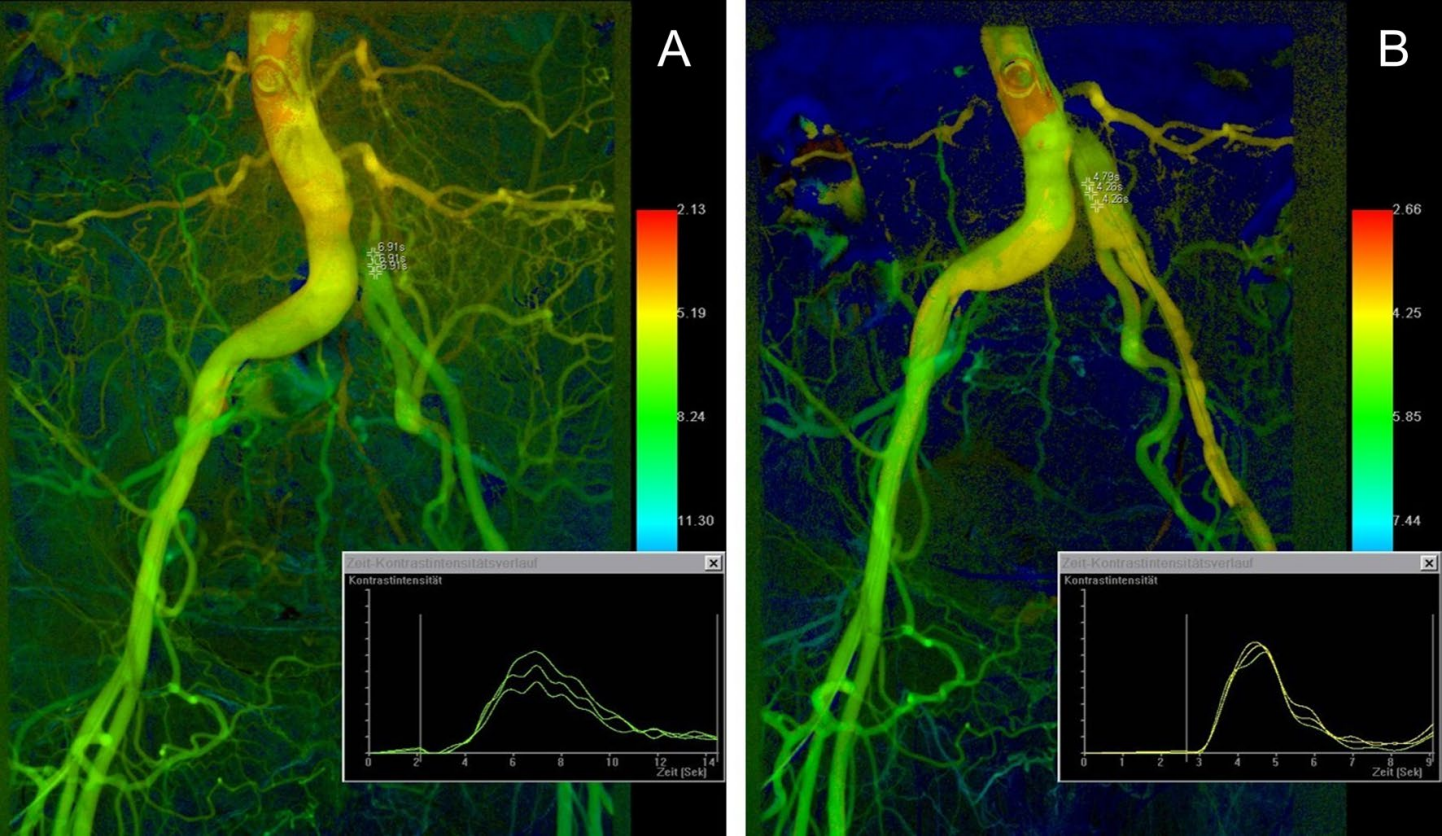
45-year-old female patient with iliofemoral malperfusion syndrome of the left leg due to acute type B aortic dissection. **a** Preinterventional color-coded composite image demonstrates the dissection membrane affecting the distal abdominal aorta and extending in the left common iliac artery. Color gradient in the left proximal iliac segment indicates compromised blood flow with colder color gradients compared to the contralateral vessel axis. Corresponding TTP was 6.91 s. **b** After creation of a fenestration window at the level above the aortic bifurcation, improvement of the color gradient is demonstrated, with a TTP value of 4.44 s. Morphological improvement of vessel anatomy is also shown. Corresponding manometry did not demonstrate an improvement of the intraluminal blood pressure gradient (prior fenestration 0 mmHg; following fenestration 3 mmHg)

The region of interest used in our study was determined by the region of malperfusion and subsequent fenestration site. The ROI placement was identical prior to and after fenestration. Each ROI was averaged out of three pixel markers (Fig. 1). In most cases, the ROI was placed in the true aortic lumen closely distal to the fenestration window. In patients with two or more fenestration windows, the aortic lumen directly downstream the fenestration tears was selected for the ROI. In cases with iliofemoral malperfusion syndrome and placement of aortic fenestration window just above the iliac bifurcation, the proximal iliac artery close to the fenestration window was the preferred vessel of interest (Fig. 2). Two radiologists (R.K., A.M.A.) agreed upon the representative ROIs. TTP data were imported into Excel (Microsoft Corp., Redmond, Washington). Differences of TTP (dTTP) prior to and following the treatment were evaluated. In the cases in which manometry data were available, differences in systolic blood pressure gradients between the true and false lumen before and after the dissection flap fenestration were analyzed. The relation between the changes of the mean systolic blood pressure gradient and dTTP was assessed. Furthermore, the presence of different outcomes regarding TTP and blood pressure gradients in Stanford type A and B dissections was analyzed. Gained data were collected with Excel (Microsoft Office 365 ProPlus, Version 1803).

### Statistical analysis

Statistical analysis was performed using a dedicated software (R®, Version 4.0.2, The R Foundation for Statistical Computing, Wien, Austria). Descriptive data are presented as means ± standard deviation (SD) for normally distributed variables or medians with ranges for non-normalized variables, if appropriate; categorical data are expressed as counts and percentages with n (%). With regard to the assessment of normality, the Shapiro test was used, rejecting the hypothesis of normality when the p-value was less than or equal to 0.05. Intergroup differences were calculated using the Mann–Whitney U test and unpaired Student´s t test, differences before and after the procedure using the Wilcoxon or paired Student´s t test. Correlation analysis of ordinal and metrical data was performed with the test according to Spearman for non-normalized variables.

## Results

**Fig. 3:**
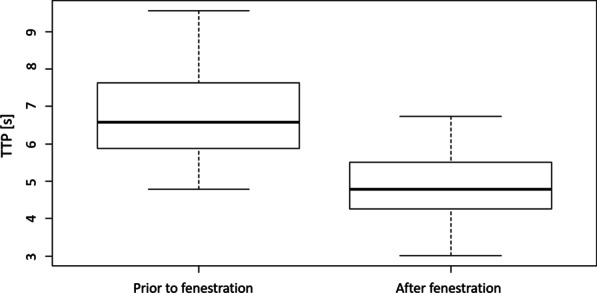
TTP (s) prior to and following endovascular dissection flap fenestration

The mean aortic TTP prior to fenestration was 6.85 ± 1.35 s. Postinterventional, TTP values were shortened with significance, resulting in a mean TTP of 4.96 ± 0.94 s (p < 0.001) and a mean dTTP of 1.89 ± 1.43 s (Fig. 3).

**Fig. 4:**
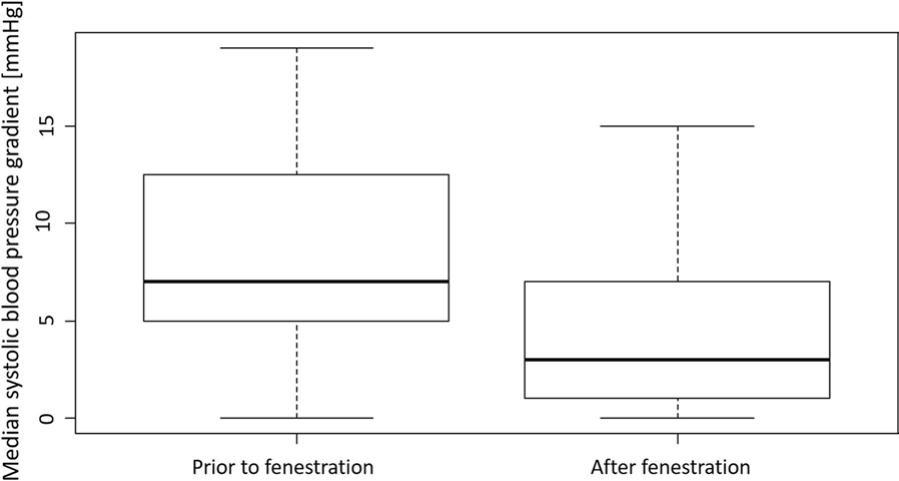
Median systolic blood pressure gradient (mmHg) prior to and following endovascular dissection flap fenestration

Available systolic blood pressure gradients between the true and the false lumen were 7.00 mmHg in median before the fenestration procedure (range 0–53 mmHg). After treatment, median systolic blood pressure gradients resulted in a value of 3.0 mmHg (range 0–15 mmHg). The median gradient reduction was 4.0 mmHg, reaching statistical significance (p = 0.031; Fig. 4).

Out of the 19 available manometry results, a sufficient blood pressure equalization according to the strict criteria (systolic pressure gradient following fenestration equal to or less than 5 mmHg) was found in 11 cases. In four cases, reductions in blood pressure gradients not reaching the predefined limit were observed. In the remaining four cases, fenestration revealed paradox results with an increase of systolic blood pressure gradients after the procedure.

When comparing the systolic blood pressure measurements, a significant reduction of transluminal pressure gradients was observed following fenestration in Stanford type B dissections (p = 0.013) but not in type A dissections (p = 0.530). Regarding TTP values, both Stanford type A and B dissections revealed significant reductions after dissection flap fenestration (p = 0.001 vs. p = 0.0002).

The relationship between dTTP and decrease of systolic blood pressure gradients was moderate and not statistically significant (r = 0.226; p = 0.351). The difference of dTTP in one or multiple fenestration windows was not statistically significant (p = 0.596).

## Discussion

TTP represents the time until the maximal concentration of contrast within the region of interest and compromised blood flow due to stenosis or true-lumen collapse is likely to lead to prolonged TTP values. In this study we aimed to investigate TTP as a suitable parameter of technical success for the improvement of hemodynamics in the compromised vessel lumen after dissection membrane fenestration. This hypothesis is supported by the results of this study as treatment led to significant dTTP values.

The hemodynamic features prevailing in aortic dissections are difficult to study and remain only partially understood, to date [12]. Unlike other endovascular therapy approaches, for example, in the treatment of peripheral arteries, the diagnostic usability of monochromatic DSA series must be questioned when assessing the technical success of aortic dissection flap fenestration [13]. Intraluminal manometry, on the other hand, is an intraprocedural technique that is favorably reported in the setting of aortic dissection flap fenestration as a potential solution for this dilemma. Nevertheless, based on our experiences, we doubt the reliability of this tool when assessing the severity of malperfusion syndromes in aortic dissections or the success of the chosen treatment, respectively. For example, even if preexisting imaging, laboratory studies and clinical presentation clearly indicated a significant true lumen collapse and compromise of perfusion to aortic branch vessels, manometry revealed no relevant transluminal pressure gradients in some cases. On the other hand, in some cases of technically feasible balloon fenestrations an improvement of pressure gradients or even worsening of those parameters was not documented. This leads us to the presumption that measurement of transluminal blood pressure gradients may be reliable only to a limited extent. The observation that manometry results and hemodynamic parameters obtained from color-coded images only showed a moderate correlation might further emphasize this limitation.

Interestingly, transluminal blood pressure gradients were differently influenced by the treatment in patients with Stanford type A and B dissections. While significant differences were documented in patients with type B dissection, patients with type A dissections presented with differences below the level of significance. An explanation for this result might be that all patients with type A dissection had undergone surgical repair treatment of the thoracic aorta prior to fenestration. Surgical repair of type A dissection seals the proximal entry of the aortic dissection, which is likely to result in alterations of hemodynamics prevailing in the distal aortic lumina. In this situation, the patency of the false lumen as well as the persisting extent of transluminal blood pressure gradient may highly depend on further existing entries and re-entries. Based on our results, we hypothesize that the parameters gained from color-coded DSA, including TTP, offer more stable and representative results in the evaluation of aortic lumen malperfusion, especially in type B dissections. All in all, both methods, blood pressure measurements as well as color-coded DSA, have weaknesses and it is unclear whether manometry is the correct gold standard at all.

As shown in another study, the presented algorithm may serve as a periprocedural success control and might also influence the treatment strategy [11]. For example, the optimal position for dissection flap fenestration remains a matter of debate. The creation of the fenestration tear at the level of the compromised vessel has been performed with remarkable success [6]. In contrast, fenestration in the proximal aorta seems to mimic an additional entry tear and may intensify the true lumen collapse. Beside the postinterventional success control, color-coded summation images might help to define the proper location and the required number of fenestrations more precisely.

To date, the utilization of quantitative color-coded DSA images has only been reported in a limited number of studies, predominantly in the field of neuroendovascular and peripheral artery procedures [8, 9, 14, 15]. Only two other studies have addressed the application of color-coding DSA in aortic dissection so far. Tinelli et al. [16] utilized parametric imaging for the assessment of chronic thoracic aortic dissections during thoracic endovascular aortic repair (TEVAR). The authors reported increased angiographic accuracy with sufficient identification of the true and false lumen in 72.7%, and identification of the entry tear in almost half of the cases. This might be particularly beneficial in the emergency setting with insufficient preoperative diagnostic management and may contribute to an optimal procedural management. Fang et al. [17] investigated the application of color-coded DSA to analyze hemodynamic changes in renal artery perfusion before and after thoracic endovascular aortic repair in type B aortic dissections. Time-intensity curve parameters, including average peak ratio, average delayed time to peak, and average area under the curve ratio, changed significantly after the procedure, suggesting the method to be beneficial in the intraprocedural evaluation of renal blood flow.

Preinterventional cross-sectional studies should be available in most cases and support the investigation of hemodynamic and morphologic features in aortic dissections. Beside conventional CTA and MRA, more novel imaging techniques, such as four-dimensional flow MRI offer more precise characterization of blood flow while also enabling the color-coding of flow velocity or other parameters [18–20]. Nevertheless, the clinical urgency of malperfusion syndromes might impede the acquisition of current imaging studies prior to the procedure, especially when being time consuming. These techniques might thus be more practicable in non-symptomatic intervals or in the setting of follow-up examinations.

Color-coded imaging gained from DSA series is an implemented algorithm, neither necessitating additional amounts of contrast media nor the use of further radiation exposure. Its application is fast and simple and thus might be feasible to be employed intraprocedurally, even within the angiography suite [21]. Therefore, the implementation of this technique in a prospective study setting would be possible. When applying color-coding to DSA, different aspects and method restrictions should be considered: Since the selection of ROIs for TTP calculation is conducted manually, ROI placement might vary among different readers, resulting in a limited interrater reliability [22]. Nonetheless, this effect might be minor as typically the same user within one procedure performs the ROI placement. Also, relevant influence of the color gradient might result from different catheter placements and the location of pixel labelling in relation to the catheter. Moreover, the amount and concentration of contrast medium as well as the imaging frame rate should be the same with regard to all DSA series. Finally, the extent of true lumen compromise might fluctuate due to dynamic flap obstruction and be furthermore influenced by different parameters, such as the aortic diameter, patients´ heart rate and blood pressure.

The presented study underlies different limitations. First, the study was conducted in a retrospective design and the number of included patients is limited. Therefore, it must be recognized that although this study showed significant TTP changes before and after fenestration, the relationship between TTP and actual hemodynamic parameters is still unclear. Second, intraprocedural manometric measurements were introduced after the start of the study, and corresponding pressure gradients were available in 19 of 25 interventions. Third, catheter placement for manometry was not necessarily the same as placement of ROIs for hemodynamic analysis, which possibly might have influenced the measurement results.

In conclusion, hemodynamic parameters extracted from color-coded DSA images may help to evaluate the procedures´ technical success in percutaneous fenestration of dissection flaps. Color-coded DSA images may offer a stable diagnostic tool, especially in cases of technically unfeasible or inconclusive manometry. However, the correlation with results from transluminal blood pressure measurements is limited. It is important to note, that due to the underlying technique, changes in cardiac output or blood pressure as well as the injection rate of contrast administration may lead to deviations.

## Data Availability

The data used and analyzed during the current study are available from the corresponding author on reasonable request.

## Abbreviations

AUC: Area under the curve
DSA: Digital subtraction angiography
CTA: Computed tomography angiography
MRA: Magnetic resonance angiography
PACS: Picture archiving and communication system
PTA: Percutaneous transluminal angioplasty
ROI: Region of interest
SFA: Superior femoral artery
TEVAR: Thoracic endovascular aortic repair
TTP: Time to peak
